# Impact of frailty on mortality, hospitalization, cardiovascular events, and complications in patients with diabetes mellitus: a systematic review and meta-analysis

**DOI:** 10.1186/s13098-024-01352-6

**Published:** 2024-05-28

**Authors:** Zhiying Miao, Qiuyi Zhang, Jijing Yin, Lihua Li, Yan Feng

**Affiliations:** 1 Jinan Maternal and Child Health Care Hospital, Jinan, Shandong China; 2 Jinan Lixia District People’s Hospital, 73 Wenhua East Road, Lixia District, Jinan, 250011 Shandong China

**Keywords:** Frailty, Diabetes, Mortality, Hospitalization, Systematic review, Meta-analysis

## Abstract

**Background:**

Several studies have focused on the impact of frailty on the health outcomes of individuals with diabetes mellitus (DM). This meta-analysis aims to systematically synthesize the existing evidence on frailty and its association with mortality, hospitalizations, cardiovascular diseases, and diabetic complications in DM.

**Methods:**

A comprehensive search in PubMed, Embase, and SCOPUS was carried out to identify relevant studies assessing the impact of frailty on mortality, hospitalizations, complications, and cardiovascular events in individuals with DM. The quality of the included studies was evaluated using the New Castle Ottawa Scale.

**Results:**

From the 22 studies included, our meta-analysis revealed significant associations between frailty and adverse outcomes in individuals with DM. The pooled hazard ratios for mortality and frailty showed a substantial effect size of 1.84 (95% CI 1.46–2.31). Similarly, the odds ratio for hospitalization and frailty demonstrated a significant risk with an effect size of 1.63 (95% CI 1.50–1.78). In addition, frailty was associated with an increased risk of developing diabetic nephropathy (HR, 3.17; 95% CI 1.16–8.68) and diabetic retinopathy (HR, 1.94; 95% CI 0.80–4.71).

**Conclusion:**

Our results show a consistent link between frailty and increased mortality, heightened hospitalization rates, and higher risks of cardiovascular disease, diabetic nephropathy, and diabetic retinopathy for patients with DM.

*PROSPERO Registration Number*: CRD42023485166

**Supplementary Information:**

The online version contains supplementary material available at 10.1186/s13098-024-01352-6.

## Introduction

The global prevalence of diabetes mellitus (DM) is on an alarming rise, and projections indicate that it will affect approximately 300 million individuals by the year 2025 [1]. As the number of patients with DM surges, the central focus of DM management remains to prevent vascular complications and preserve the quality of life (QOL) for affected individuals [2, 3]. The significance of effective DM treatment extends beyond symptom management; it plays a pivotal role in improving prognoses and, crucially, in averting the onset of cardiovascular diseases—a leading cause of morbidity and mortality among individuals with DM [4, 5].

The escalating incidence of hospitalizations among patients with DM due to complications and severe hypoglycemia has introduced a new dimension to the landscape of DM management [6, 7]. Hospitalizations reflect immediate health risks and amplify medical expenses, underscoring the imperative to control healthcare costs associated with DM-related admissions.

Frailty represents a condition of physical and mental weakness that can develop in aging individuals [8]. Interventions targeting frailty have shown promise in preserving activities of daily living and enhancing the QOL of affected individuals. The incidence of frailty among middle-aged to elderly patients with DM ranges from 32 to 48% [9, 10].

While classical risk factors such as hypertension, dyslipidemia, and smoking habit contribute to approximately 60% of deaths and cardiovascular diseases in patients with DM, frailty has emerged as a significant factor in the remaining cases [11–13]. Additionally, frailty is linked to heightened hospitalization rates. Thus, early detection of frailty, a modifiable risk factor, holds substantial clinical importance.

The present systematic review pools the evidence of the association between frailty and critical health outcomes, including mortality, hospitalization rates, complications, and cardiovascular events in patients with DM.

## Methods

This systematic review and meta-analysis was carried out in accordance to PRISMA guidelines [14] and registered the protocol at PROSPERO (registration number CRD42023485166). The review was in line with the registered protocol and did not deviate.

### Search strategy

Major electronic databases, including PubMed, Embase, and SCOPUS were searched. The search strategy combined keywords and Medical Subject Heading (MeSH) terms related to DM, frailty, mortality, hospitalization, complications, and cardiovascular events. The search string included the following terms:

(Frailty OR frail OR ‘‘Frail Elderly’’ OR ‘‘Frailty Syndrome’’) AND (Diabetes OR ‘‘Diabetes Mellitus’’ OR diabetic) AND (Mortality OR death) AND (Hospitalization OR ‘‘Hospitalizatio’’ OR admission OR inpatient) AND (Complications OR ‘‘Complications’’ OR ‘‘Adverse Outcomes’’) AND (Cardiovascular Events OR ‘‘Cardiovascular Diseases’’ OR ‘‘Cardiac Event’’).

The last electronic search was carried out on the 15th of December, 2023. The details of the search in specific databases are provided. (Supplementary Table 1).

The reference lists of included articles and relevant reviews were screened, and a manual search of issues of pertinent diabetology journals was carried out for any potentially eligible studies that might have been missed with the digital searches.

The citations were imported to a digital citation manager software (EndNote version 20, Clarivate Analytics, USA) to identify the duplicates across the three databases and get them removed.

### Eligibility criteria

The observational studies like cohorts, cross-sectional and case–control studies, and clinical trials assessing frailty's impact on mortality, hospitalization, complications, and cardiovascular events in individuals with DM, employing recognized frailty assessment tools or criteria and published in the English language, were deemed eligible.

Non-human studies, case reports, editorials, and conference abstracts without full-text availability were excluded from this review.

### Study selection process

Two independent reviewers screened the search-identified studies based on titles and abstracts for potential relevance to the study objectives. Inclusion and exclusion criteria were applied to select studies on individuals diagnosed with DM, which applied recognized frailty assessment tools and reported outcomes related to mortality, hospitalization, complications, and cardiovascular events. After the initial screening, the researchers conducted a full-text review. They independently assessed the full text of selected studies to determine their eligibility for inclusion. Any discrepancies or disagreements between the two reviewers were resolved through discussion and consensus with the help of a third reviewer.

### Data extraction

A standardized data extraction form was generated using MS Excel Spreadsheet (Microsoft, USA) to collect relevant information from selected studies systematically. The form included fields for study characteristics (author, publication year), participant demographics, study design, frailty assessment methods, mortality-related outcomes, hospitalizations, complications, and cardiovascular events.

### Quality of included studies

Newcastle–Ottawa Scale (NOS) was used to systematically assess the quality and risk of bias in the included studies and enhanced the synthesized evidence's reliability. The scale encompassed three key components (study group selection, group comparability, and outcome ascertainment) with specific criteria, such as the representativeness of the exposed cohort, comparability based on design or analysis, and reliable ascertainment of outcomes. Each criterion was assigned a star rating and an overall score was obtained to indicate the study's quality.

### Data synthesis

The characteristics and key findings of the included studies was summarized in a narrative synthesis, and the meta-analysis was carried out using RevMan 5.4 v (Cochrane Collaboration, UK) with data from studies deemed suitable for statistical pooling [15, 16]. A random-effects model was used to combine data from individual studies to derive an overall quantitative estimate of the impact of frailty on health outcomes in individuals with DM. We calculated pooled estimates, including hazard ratios (HRs) and odds ratios (ORs), and plotted them as forest plots. Heterogeneity among studies was assessed using the I^2^ statistic. We considered an I^2^ statistic value higher than 70% highly heterogeneous and one lower than 50% indicative of low heterogeneity and between 50 and 70% as moderate heterogeneity. Subgroup analyses were carried out to explore variations based on study design, frailty assessment tools, and other relevant factors. We assessed publication bias using funnel plots to enhance the reliability of the synthesized evidence.

Finally, the results were interpreted in terms of both the narrative and quantitative syntheses in the context of the study’s objectives, acknowledging limitations and providing recommendations for future research.

## Results

**Fig. 1:**
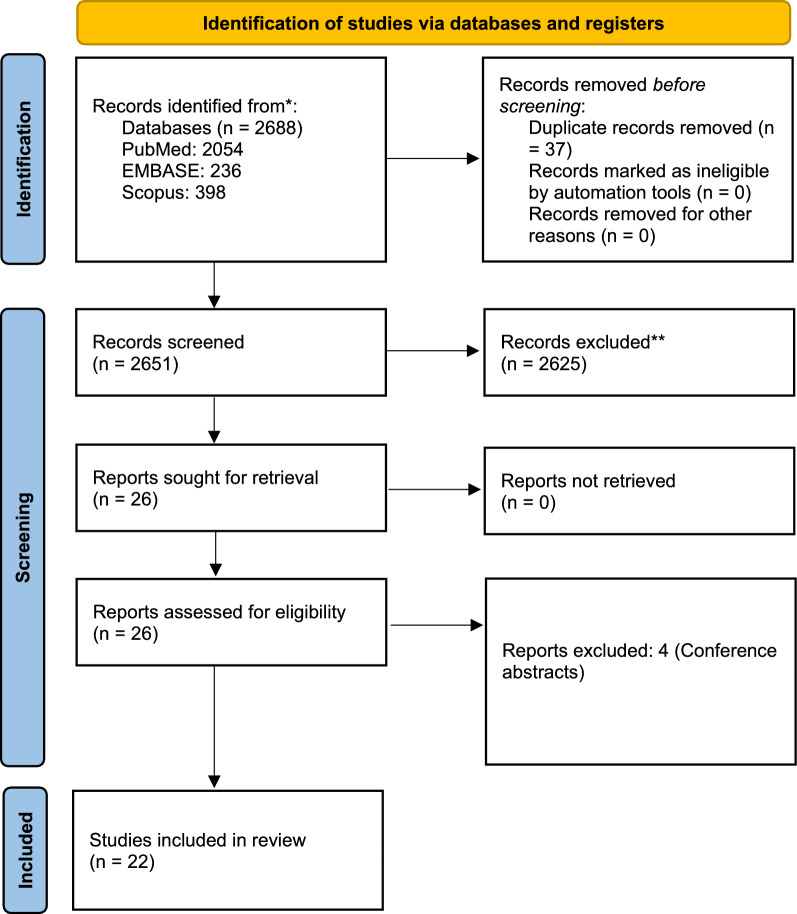
Study selection flow chart

A total of Twenty-two studies were included in this review. [17–38] The search was conducted comprehensively to identify 2688 records. After removing 37 duplicates, we screened 2651 records based on title and abstract for relevant studies. Out of these 2651 records, only Twenty-six records were subjected to full-text analysis to match the selection criteria. Finally, Twenty-two studies were included that satisfied the eligibility criteria (Fig. 1). Four records were excluded, and the reason for exclusion was provided (Supplementary Table 2).

### Characteristics of included studies

The included studies were conducted in settings such as Taiwan, China, the UK, Brazil, the USA, Japan, Singapore, Spain, and Italy, and they were based on a range of study designs such as five population-based longitudinal studies [18, 22, 26, 32, 35] five retrospective cohort studies [19, 21, 27, 29, 30] seven prospective studies [20, 23, 25, 28, 33, 34, 37] two clinical trials [24, 36], and three cross-sectional analyses [17, 31, 38]. Sample sizes varied widely, from smaller cohorts with hundreds of participants to massive studies involving over half a million individuals (Table 1).

**Table 1:** Characteristics of included studies

Authors	Year	Location	Study design	Sample size	Age	Male%	Female%	Frailty Index	Frail (%)	Outcomes	Follow-up (years)	Adjusted for
Wang et al. [17]	2023	China, Mainland	Cross-sectional study	168	> 65 years	48.2%	51.8%	FRAIL scale, including 5 components: Fatigue, Resistance, Ambulation, Illness, and Loss of weight	22.6%	Mortality, Hospitalization, Emergency, and Clinic visit	1	Sex, age, BMI, Insulin-dependency, HbA1c, and co-morbidities
Huang et al. [18]	2023	Taiwan	Population-based longitudinal study	123,172	60.68 years	NR	47.30%	Multimorbidity frailty index	NR	Mortality, unplanned hospitalisation, cardiovascular disease-related mortality, major adverse cardiovascular events, DM-related hospitalisation and hypoglycemia	10	Age, sex, number of anti-diabetic medications and metformin use during follow-up
Weng et al. [19]	2023	Taiwan	Retrospective longitudinal cohort study	921	83.2	73.80%	NR	Modified Rockwood frailty index	79.30%	All-cause mortality	2.92	Confounders, including kidney function and other diabetic complications
Mickute et al. [20]	2023	UK	Population-based prospective study	397,254	NA	NR	NR	Concordance index (Harrell’s C-index)	NR	Mortality	12.5	Age and sex
Lin et al. [21]	2023	China, Mainland	Single center retrospective study	293	72 (10)	51.20%	48.80%	Laboratory frailty index	17.80%	Diabetic nephropathy; diabetic retinopathy; coronary artery disease; cerebrovascular disease; peripheral arterial disease; diabetic peripheral neuropathy; activities of daily living	5	NA
Wu et al. [22]	2022	UK	Population-based Prospective longitudinal cohort study	18,062	59.4 ± 7.2 years	62.60%	37.40%	NR	10.40%	Primary outcome: diabetic microvascular complications; Secondary outcomes: Diabetic nephropathy, Diabetic neuropathy, and Diabetic retinopathy	12	Age, sex, ethnicity, educational attainment, Townsend deprivation index, annual household income, assessment centres, smoking status, alcohol intake, healthy diet score, BMI, number of long-term conditions, DM duration, HbA1c, DM medication use, lipid-lowering treatment, antihypertensive medication use, and aspirin use
He et al. [23]	2022	China, Mainland and UK	Prospective cohort study	7933	60	NR	52.2%	Frailty index	NR	Progression of pre-DM to DM, cardiovascular disease, and all-cause mortality	4	Age, sex, education, marital status, drinking status, smoking status, body mass index, systolic blood pressure, triglyceride, high-density lipoprotein, cholesterol, glycated haemoglobin, and C-reactive protein
Espeland et al. [24]	2022	USA	Multisite, single-blind randomized controlled clinical trial	3842	45–76 years	NR	NR	Multimorbidity index and frailty index	NR	Cancer, cardiac arrhythmia, chronic kidney disease, congestive heart failure, coronary artery disease, depression, dyslipidaemia, hypertension, stroke, and mortality	8	Gender, current age, education, race/Ethnicity, and randomization assignment, number of prior cognitive assessments, and baseline BMI, multimorbidity, and frailty index
Akan et al. [25]	2022	Brazil	Single center cohort study	100	> 65 years	66%	34%	Edmonton, Frail, and Prisma-7 scores	NR	Mortality and hospitalization	6 months	NR
Leung et al. [26]	2021	USA	Longitudinal population-based study	884	57–91 years	NR	NR	NR	25%	frailty, disability, and 5-year mortality	5	Age and gender
Presley et al. [27]	2019	USA	Retrospective cohort study	495	65 (58, 75)	99%	NR	Modified frailty index	59%	Mortality	NA	Demographic, administrative, and clinical electronic health records
Kitamura et al. [28]	2019	Japan	Prospective, community-based study	1271	71.0 ± 5.6 years	42.80%	57.20%	NR	12%	Frailty, DM, all-cause mortality and incident disability	8.1	Age, sex, hypertension, high total cholesterol, low total cholesterol, low estimated glomerular filtration rate, overweight, low body mass index, anemia, hypoalbuminemia, low mini-mental state examination score, history of stroke
Ferri-Guerra et al. [29]	2020	USA	Retrospective cohort study	763	59.4 ± 7.2 years	98.30%	NR	frailty index	50.50%	Frailty, DM, all-cause hospitalizations and mortality	561 days	Age, race, ethnicity, median income, history of hospitalizations, comorbidities, duration of DM and glycemic control
Chao et al. [30]	2018	Taiwan	Retrospective longitudinal cohort study	560,795	56.4 ± 13.8 year	NR	46.10%	NR	0.30%	Hospitalization or intensive care unit, mortality and incident cardiovascular events	3.14	Demographic profiles, comorbidities, DM severity, and medications
Li et al. [31]	2018	Taiwan	Cross-sectional study	719	≥ 65 years	NR	58%	NR	9.40%	Frailty, all-cause hospital admission and emergency	NA	Age, sex, education, marital status, duration of DM, use of insulin, falls, activities of daily living disability, and instrumental activities of daily living disability
Thein et al. [32]	2018	Singapore	Population-based longitudinal study	486	67.3 ± 7.5 years	NR	59.50%	NR	4.70%	Disability, frailty and mortality	11	Sex, age, education, smoking, alcohol intake, physical activity and BMI
Castro-Rodriguez et al. [33]	2016	Spain	Prospective cohort study	363	NR	NR	NR	Frailty Trait Scale, Rockwood Frailty Index	NR	All-cause deaths and Functional disability	Mortality = 5.5, Disability = 4.98	Age, sex, a measure of disease burden and FI
Chode et al. [35]	2016	USA	Population-based longitudinal cohort study	222	57.43 ± 4.4 years	NR	69.80%	International Academy of Nutrition and Aging frailty scale, study of osteoporotic fractures frailty scale, cardiovascular health study frailty scale, and frailty index	NR	Activities of daily living, instrumental activities of daily living, lower body functional limitations, short physical performance battery, one-leg stand, and grip strength and frailty	9	Age and gender
Liccini et al. [34]	2016	Missouri	Observational study	198	64.9 ± 8.7 years	52.50%	47.50%	Frailty index	28.80%	Self-reported activities of daily living, hospitalization and Mortality	6 months	Age, sex, education, and HbA1C
Li et al. [36]	2015	China, Mainland	Single center pilot study	146	80 years	NR	21.90%	FRAIL scale	15.10%	Macroangiopathy, nephropathy, hospitalizations and mortality	2	Age, gender, cognition, BMI, and duration and severity of DM
Wang et al. [37]	2014	USA	Cohort study	2415	73.68 ± 5.25 years	98%	NR	NR	NR	All-cause mortality	5.30 ± 2.39	Propensity score of metformin use and covariates: age, race/ethnicity, DM duration, Charlson comorbidity score, statin use, smoking status, BMI, LDL, and HbA1c
Cacciatore et al. [38]	2013	Italy	Cross-sectional study	1288;	74.2 ± 6.3 years	43%	57%	Frailty staging system	41.30%	Disability, frailty and mortality	12	Sex, age and other several variables including the associations between frailty and DM. and frailty and sex

*NR* not reported, *NA* not available, *UK* United Kingdom, *HbA1C* glycated hemoglobin; *BMI* body mass index, *DM* diabetes mellitus, *FI* frailty index, *LDL* low-density lipoprotein

The frailty assessment tools used in these studies demonstrated a breadth of approaches, with indices like the multimorbidity frailty index (MFI), laboratory frailty index (LFI), and modified Rockwood frailty index (RFI), reflecting the multifaceted nature of these evaluations. Notably, the prevalence of frailty among study populations exhibited considerable diversity, with some studies reporting high percentages (e.g., 79.30% in Weng et al. 2023) and others reporting lower figures (Table 1).

The outcomes under investigation were extensive, ranging from immediate concerns such as mortality, hospitalization, and cardiovascular events to specific DM-related complications and functional disabilities. The follow-up durations were also diverse, spanning short-term assessments of 6 months [25] to more extended observational periods of about 12.5 years [20].

None of the studies were found to have any potential conflict of interest, and the source of funding of each study are made available (Supplementary Table 3).

Quality of included studies:

We found the quality of the included studies to be good, with NOS scores ranging between 7 and 9 (Table 2).

**Table 2:** NOS criteria assigned to included studies to assess their overall quality

Study	Year	Selection				Comparability	Outcome			Total
		Representativeness of the exposed cohort	Selection of the nonexposed cohort	Ascertainment of exposure	Demonstration of outcome of interest	Basis of the design or analysis	Assessment of outcome	Appropriate follow-up length for outcomes	Adequate follow-up	
Wang et al. [17]	2023	0	1	1	1	1	1	1	1	7
Huang et al. [18]	2023	0	1	1	1	1	1	1	1	7
Weng et al. [19]	2023	0	1	1	1	1	1	1	1	7
Mickute et al. [20]	2023	1	1	0	1	1	1	1	1	7
Lin et al. [21]	2023	1	1	1	1	2	1	1	1	9
Wu et al. [22]	2022	1	1	0	1	1	1	1	1	7
He et al. [23]	2022	1	1	1	1	1	1	1	1	8
Espeland et al. [24]	2022	1	1	1	1	1	1	1	1	8
Akan et al. [25]	2022	1	1	1	1	1	1	1	1	8
Leung et al. [26]	2021	0	1	1	1	1	1	1	1	7
Presley et al. [27]	2019	1	1	0	1	1	1	1	1	7
Kitamura et al. [28]	2019	1	1	0	1	1	1	1	1	7
Ferri-Guerra et al. [29]	2020	1	1	0	1	1	1	1	1	7
Chao et al. [30]	2018	0	1	1	1	1	1	1	1	7
Li et al. [31]	2018	1	1	1	1	1	1	1	1	8
Thein et al. [32]	2018	1	1	0	1	1	1	1	1	7
Castro-Rodriguez et al. [33]	2016	1	1	1	1	1	1	1	1	8
Chode et al. [35]	2016	1	1	1	1	1	1	1	1	8
Liccini et al. [34]	2016	1	1	1	1	1	1	1	1	8
Li et al. [36]	2015	1	1	1	1	1	1	1	1	8
Wang et al. [37]	2014	1	1	0	1	1	1	1	1	7
Cacciatore et al. [38]	2013	1	1	1	1	1	1	1	1	8

### Meta-analysis

#### Mortality

**Fig. 2:**
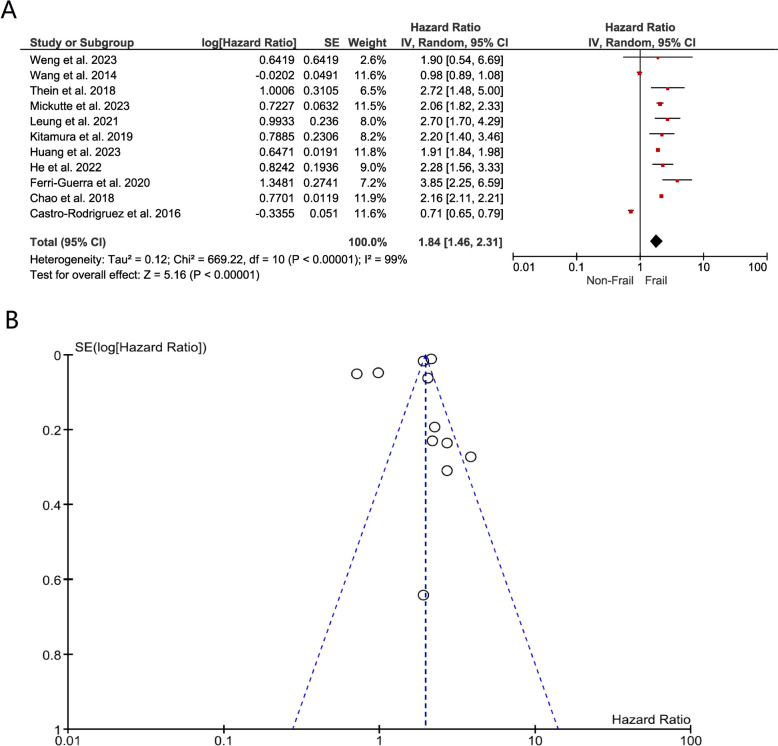
**a** Forest plot showing the pooled hazard ratio estimate for mortality in frail patients with DM; **b** Funnel plot showing distribution of the pooled hazard ratio estimate for mortality in frail patients with DM

Frailty: Our pooled hazard ratio of 1.84 (95% CI 1.46–2.31) suggests that individuals with DM and frailty have a 1.84 times higher mortality risk than those without frailty. This substantial association underscores the importance of frailty as a significant predictor of mortality in diabetic populations (Fig. 2a). The funnel plot shows most studies distributed inside the funnel, with Castro-Rodiguez et al. 2016 and Wang et al. 2014 studies falling outside, a finding suggestive of potential bias (Fig. 2b).

**Fig. 3:**
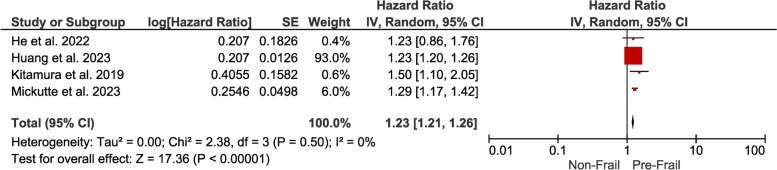
Forest plot showing the pooled hazard ratio estimate for mortality in pre-frail patients with DM

Pre-Frailty: The hazard ratio of 1.23 (95% CI 1.21–1.26) for pre-frail individuals indicates a moderate but statistically significant association with mortality. Even at the pre-frail stage, the risk of death in individuals with DM is increased (Fig. 3).

#### Hospitalization

**Fig. 4:**
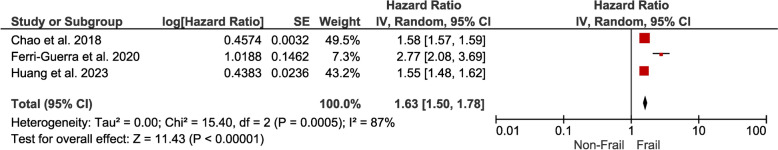
Forest plot showing the pooled hazard ratio estimate for hospitalizations in frail patients with DM

**Fig. 5:**
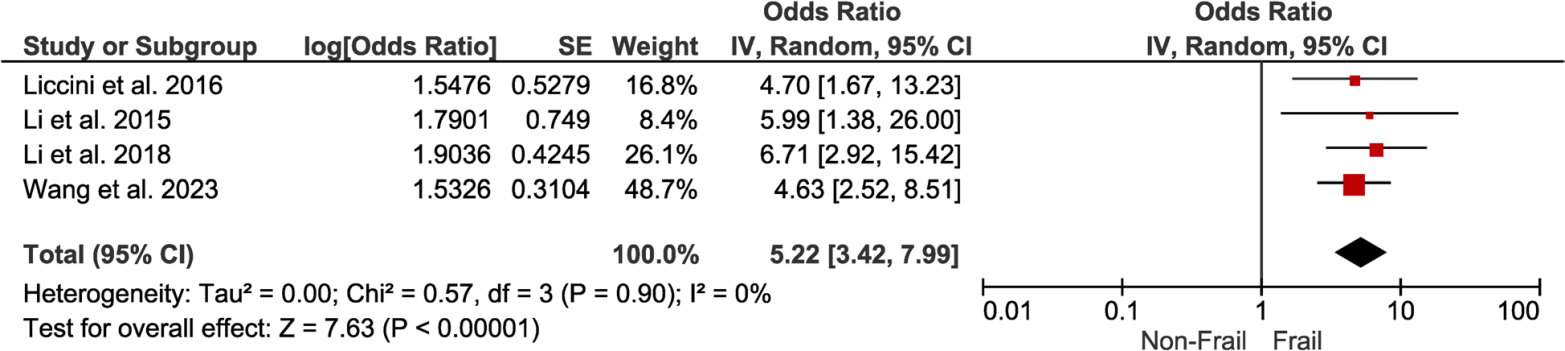
Forest plot showing the pooled odds ratio estimate for hospitalizations in frail patients with DM

*Frailty* The hazard ratio (1.63; 95% CI 1.50–1.78) and odds ratio (5.22; 95% CI 3.42–7.99) highlight a substantial increase in the risk of hospitalizations for individuals with DM and frailty. This dual perspective underscores the robustness of the association (Figs. 4,5).

**Fig. 6:**
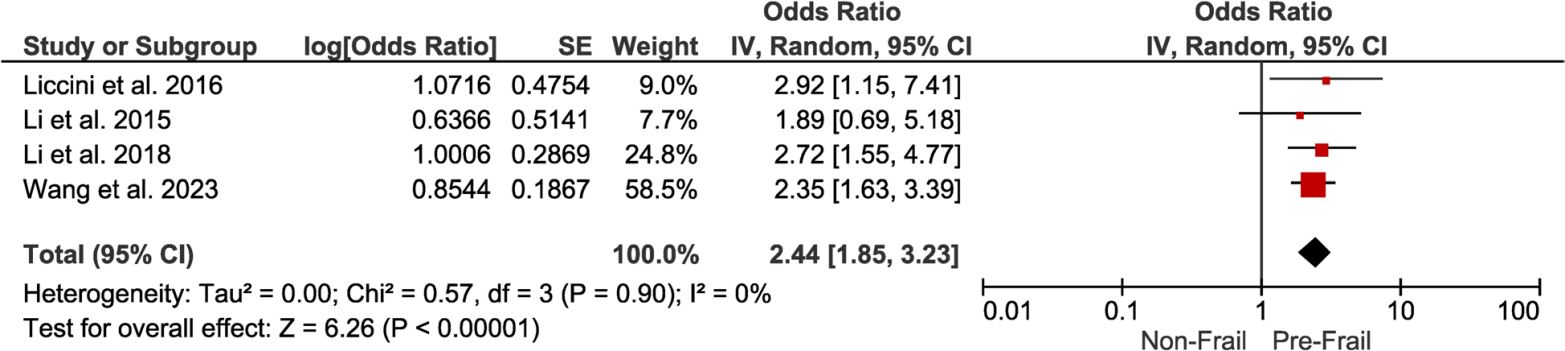
Forest plot showing the pooled odds ration estimate for hospitalizations in pre-frail patients with DM

*Pre-Frailty* The odds ratio at 2.44 (95% CI 1.85–3.23) for pre-frail individuals indicates a moderate but significant association with hospitalizations. Thus, individuals at the pre-frail stage also exhibit an elevated risk of hospitalizations (Fig. 6). *Cardiovascular Disease (CVD):*

**Fig. 7:**
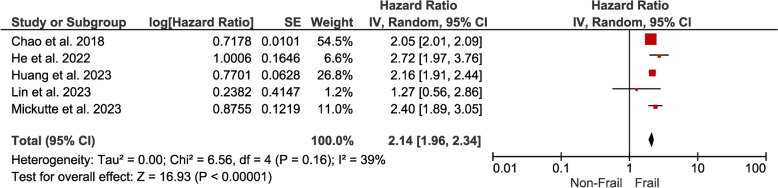
Forest plot showing the pooled hazard ratio estimate for developing CVD in frail patients with DM

*Frailty* The pooled hazard ratio of 2.14 (95% CI 1.96–2.34) means that individuals with DM and frailty face more than twice the risk of developing cardiovascular disease compared to those without frailty. This emphasizes the substantial risk posed by frailty for cardiovascular complications (Fig. 7).

**Fig. 8:**
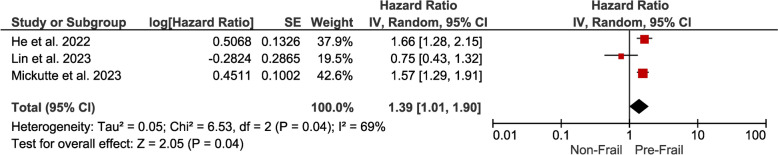
Forest plot showing the pooled hazard ratio estimate for developing CVD in pre-frail patients with DM

*Pre-Frailty* Pre-frail individuals presented a hazard ratio of 1.39 (95% CI 1.01–1.90), indicating a significant but comparatively lower association with cardiovascular disease than that of frail individuals. However, even at the pre-frail stage, there is an increased risk of CVD (Fig. 8).

#### Complications

**Fig. 9:**
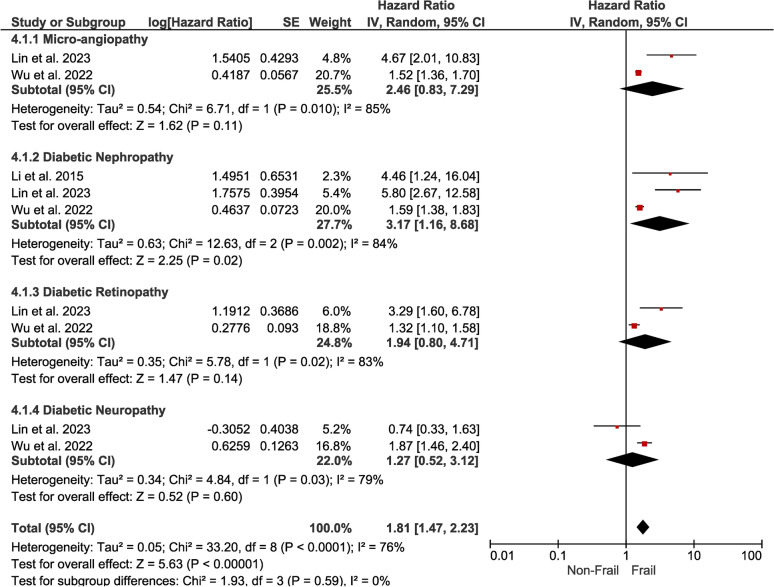
Forest plot showing the pooled hazard ratio estimate for developing various complications in frail patients with DM

*Frailty* Individuals with DM and frailty presented an increased risk of complications (HR, 1.81; 95% CI 1.47–2.23) compared to individuals with DM alone. We further explored this association in subgroup analyses for specific complications (Fig. 9).

*Micro-angiopathy* The hazard ratio of 2.46 (95% CI 0.83–7.29) suggests an increased risk; however, no association was detected, but the wide confidence interval indicates uncertainty and the need for cautious interpretation.

*Diabetic Nephropathy* The hazard ratio of 3.17 (95% CI 1.16–8.68) indicates the presence of a significant association between frailty and diabetic nephropathy, suggesting that frailty may be a predictor of kidney complications.

*Diabetic Retinopathy* The hazard ratio of 1.94 (95% CI 0.80–4.71) indicates an increased risk without statistical significance, suggesting no association, emphasizing further investigation.

*Diabetic Neuropathy* The hazard ratio 1.27 (95% CI 0.52–3.12) suggests a potential association (no statistical significance) between frailty and neuropathic complications.

**Fig. 10:**
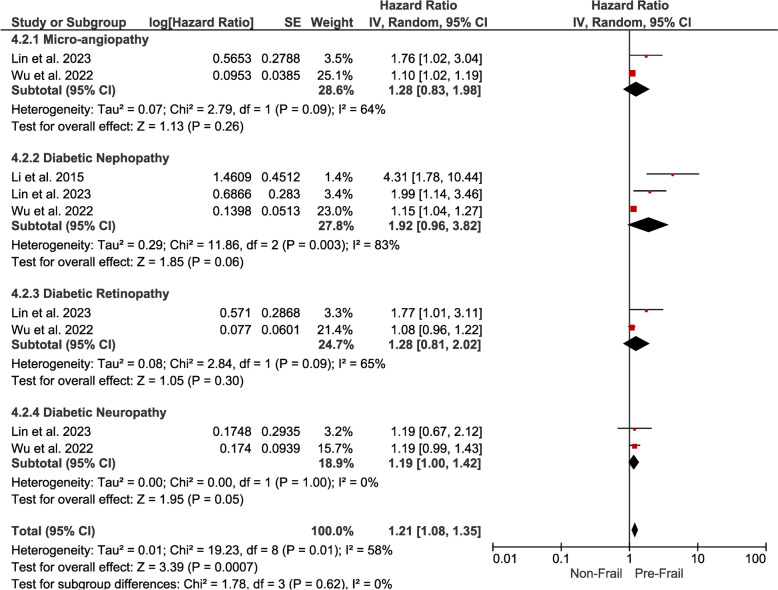
Forest plot showing the pooled hazard ratio estimate for developing various complications in pre-frail patients with DM

*Pre-Frailty* Pre-frail individuals with DM presented a hazard ratio of 1.21 (95% CI 1.08–1.35) for overall complications, indicating a moderate but significant association. Subgroup analyses revealed some associations for specific complications (Fig. 10).

*Micro-angiopathy* The hazard ratio 1.28 (95% CI 0.83–1.98) indicates a potential association without statistical significance.

*Diabetic Nephropathy* The hazard ratio 1.92 (95% CI 0.96–3.82) suggests a potential association without statistical significance.

*Diabetic Retinopathy* The hazard ratio 1.28 (95% CI 0.81–2.02) indicates a potential association without statistical significance.

*Diabetic Neuropathy* The hazard ratio 1.19 (95% CI 1.00–1.42) indicates a moderate and statistically significant association between pre-frailty and neuropathic complications.

### Asymmetry of evidence

The funnel plots were assessed for the plots to find the symmetrical distribution of included studies of all outcomes suggestive of no publication bias.

## Discussion

This systematic review and meta-analysis aimed to pool the evidence for associations between frailty and health outcomes (specifically for mortality, hospitalizations, complications, and cardiovascular events) in individuals with DM. Our results underscore the significance of frailty as a critical factor influencing the health trajectory of patients with DM.

The use of diverse frailty indices by different studies, including the multimorbidity frailty index [39], laboratory frailty index [40], modified Rockwood frailty index [41], and others [42], underscores the multidimensional nature of frailty assessments. The prevalence of frailty varied across studies, with rates ranging from 10.40% to 79.30%, demonstrating heterogeneity in frailty representation.

Our quantitative synthesis revealed a heterogeneous landscape of results across the included studies. The meta-analysis demonstrated a statistically significant association between frailty and adverse health outcomes; in particular, frail individuals with DM presented heightened mortality and increased rates of hospitalizations and complications.

The observed hazard ratios for both frailty and pre-frailty indicate a significantly increased risk of mortality in individuals categorized as frail or pre-frail. This finding aligns with others emphasizing frailty as a crucial predictor of overall mortality. The robustness of this association, evidenced by the synthesis of data from multiple studies, demonstrates the clinical relevance of frailty assessments for life expectancy predictions.

Previous meta-analyses [10, 43] have explored the associations between frailty and health outcomes in individuals with DM, providing valuable insights. However, this current meta-analysis contributes to the existing literature by incorporating the latest studies and expanding the scope to include a comprehensive assessment of individual associations with mortality, hospitalizations, cardiovascular disease, and diabetic complications. Notably, our review provides updated effect estimates that clarify the effects of frailties on specific outcomes.

The mechanisms linking frailty to poor outcomes in individuals with DM are complex and multifaceted. Frailty may exacerbate the challenges posed by DM through various pathways, including inflammation, hormonal dysregulation, and impaired physiological reserves [44, 45]. Frail individuals may experience difficulties managing DM-related self-care tasks, leading to poor glycemic control [9, 46]. Additionally, the inflammatory state associated with frailty may contribute to the progression of diabetic complications, further compromising an individual’s overall health. Frail individuals exhibit a substantially elevated risk of hospitalization, as indicated by both hazard and odds ratios. The magnitude of the association emphasizes the vulnerability of frail individuals to health events necessitating hospital care. Our findings demonstrate the importance of identifying and managing frailty as a preventive measure to reduce the burden on healthcare systems.

The association between frailty and the development of cardiovascular diseases aligns with the growing recognition of frailty as a cardiovascular risk factor. The increased hazard ratio for frailty and CVD emphasizes the need for integrated cardiovascular care in individuals identified as frail [47]. Moreover, the association with pre-frailty suggests that prompt interventions may mitigate cardiovascular risks.

We found a significant association between frailty and an elevated risk of complications in individuals with DM, including microangiopathy, diabetic nephropathy, diabetic retinopathy, and diabetic neuropathy. The nuanced findings suggest that frailty is a general predictor of complications that may also contribute specifically to diabetic complications. Thus, tailored interventions addressing frailty in DM care are essential [9, 22]. Our analysis revealed a significant hazard ratio for microangiopathy in frail individuals, indicating a higher risk of microvascular complications. Microangiopathy is a hallmark of DM, involving damage to small blood vessels, leading to complications such as nephropathy, retinopathy, and neuropathy. The association between frailty and microangiopathy emphasizes the need for an integrated approach to managing frailty and DM to prevent microvascular complications. Frailty was also notably associated with an increased hazard ratio for diabetic nephropathy, underscoring the vulnerability of frail individuals to renal complications. The kidneys' microvasculature is particularly susceptible to DM damage, leading to nephropathy [48, 49]. Recognizing this heightened risk in frail individuals is important for early detection and interventions that mitigate the progression of diabetic nephropathy.

Diabetic retinopathy is a sight-threatening complication resulting from damage to the blood vessels in the retina [50]. The association of diabetic retinopathy with frailty suggests that individuals with frailty may be at a higher risk of developing severe eye complications. The presence of frailty emphasizes the need for regular ophthalmological screening and targeted interventions to prevent or manage this severe complication.

Frailty is associated with an increased hazard ratio for diabetic neuropathy, probably due to the susceptibility of frail individuals to nerve damage. Diabetic neuropathy can lead to pain, numbness, and a range of sensory and motor deficits [51]. Our findings suggest that frail individuals with DM may experience more neurological complications than their non-frail counterparts, emphasizing the need for early detection and multidisciplinary management to prevent or ameliorate diabetic neuropathy.

These findings collectively highlight the multifaceted impact of frailty on the health outcomes of individuals with DM. The increased mortality, hospitalization, cardiovascular disease, and complications risks emphasize the need for comprehensive frailty assessments and targeted interventions to improve outcomes for individuals with DM, especially for those identified as frail or pre-frail.

## Strength and limitations

The strength of this review lies in its comprehensive approach to investigating the prognostic impact of frailty in patients with diabetes mellitus. By systematically analyzing a wide range of outcomes including mortality, hospitalization, cardiovascular events, and complications, this review provides a thorough understanding of the implications of frailty in this population. The inclusion of a large number of studies and participants enhances the generalizability of the findings, while the rigorous statistical methods employed ensure robustness and reliability. Furthermore, the meticulous assessment of heterogeneity and potential sources of bias adds to the credibility of the results.

To our finding, the funnel plot depicted the symmetrical distribution of studies within the limits of funnel, suggesting no publication bias among the included studies. However, the methodological diversity among the included studies resulted in challenges for our analysis. The studies lying out of the funnel plot for the forest plot assessing mortality could be affected by the small study effect. The same could be further assessed by sensitivity analysis to clearly find out whether such study have an enormous effect. Variations in study design, frailty assessment tools, and outcome measures contributed to heterogeneity. Frailty assessment tools ranged from laboratory-based indices to self-reported scales. This heterogeneity introduced variability in the definition and identification of frailty, hindering our findings' generalizability. The included studies also had different factors adjusted for in their analyses, potentially leading to heterogeneity. We identified high-quality studies through our comprehensive assessment based on the Newcastle–Ottawa Scale. One limitation of our review is the lack of prior elaboration on the stratification of the population into frail and prefrail categories, which may have led to inconsistency across different frailty scores. This lack of clarity in categorization could potentially introduce bias and affect the interpretation of the results.

Clarifying the impact of frailty on health outcomes in DM has profound clinical implications. Our findings underscore the need for a comprehensive approach to DM management that incorporates frailty assessments as a routine component. Identifying frailty early in the course of DM may guide tailored interventions to prevent complications, reduce mortality, and optimize the quality of life for affected individuals. Clinicians should consider integrating frailty assessments using validated tools into routine DM care.

By specifically focusing on individuals with DM, our study offers valuable insights into the prognostic impact of frailty within this specific patient cohort. This targeted approach enables clinicians to gain a deeper understanding of the complex relationship between frailty and DM, thereby facilitating more personalized and effective patient care strategies.

Our results lay the foundation for future studies. Given the complexity of frailty and its multifaceted impact on health outcomes in DM, prospective studies exploring the temporal association between frailty and DM-related events are crucial. Longitudinal designs incorporating repeated frailty assessments can offer insights into the dynamic nature of frailty and its implications over time. A meta-regression analysis analytical approach could be carried out in future for elucidating the sources of heterogeneity observed in the pooled evidence, thereby strengthening the robustness of our findings. Additionally, comparative effectiveness studies evaluating the efficacy of different frailty interventions in diabetic populations will contribute to evidence-based guidelines for clinical practice.

## Conclusion

This comprehensive meta-analysis underscores the significant association between frailty and adverse outcomes in individuals with DM. The robust synthesis of data from diverse studies across multiple countries and designs revealed a consistent link between frailty and increased mortality, heightened hospitalization rates, and a higher risk of cardiovascular disease. Moreover, our subgroup analyses showed the specific associations between frailty and diabetic complications, emphasizing the need for an integrated approach to patient care. These findings are essential for clinical practice and highlight the need for routine frailty assessments in individuals with DM.

### Supplementary Information

Supplementary Material 1.

## Data Availability

The datasets used and analyzed during the current study are available from the corresponding author upon reasonable request.
