# Supplementary material for: Impact of frailty on mortality, hospitalization, cardiovascular events, and complications in patients with diabetes mellitus: a systematic review and meta-analysis
Source: Diabetol Metab Syndr. 2024 May 28;16:116. doi: 10.1186/s13098-024-01352-6 (PMC11131325; doi:10.1186/s13098-024-01352-6)
Supplement: Supplementary file 1 — Supplementary Material 1. [file 13098_2024_1352_MOESM1_ESM.docx]

Supplementary Table 1: Search strategy of specific databases

| Database | Search String | Records |
| --- | --- | --- |
| PubMED | (Frailty OR frail OR "Frail Elderly" OR "Frailty Syndrome") AND (Diabetes OR "Diabetes Mellitus" OR diabetic) | 2054 |
| SCOPUS | TITLE-ABS-KEY ("frailty" OR "frail older adults" OR "geriatric frailty" OR "physical frailty") AND TITLE-ABS-KEY ("diabetes" OR "diabetes mellitus" OR "type 2 diabetes" OR "diabetic" OR "hyperglycemia") | 398 |
| EMBASE | (frailty OR "frail older adults" OR "geriatric frailty" OR "physical frailty") AND (diabetes OR "diabetes mellitus" OR "type 2 diabetes" OR diabetic OR hyperglycemia) AND (Mortality OR death) AND (Hospitalization OR "Hospitalization" OR admission OR inpatient) AND (Complications OR "Complications" OR "Adverse Outcomes") AND (Cardiovascular Events OR "Cardiovascular Diseases" OR "Cardiac Events") | 236 |

Supplementary Table 2: List of excluded studies

| **Sl.No.** | **Studies** | **Reason for Exclusion** |
| --- | --- | --- |
| 1 | Wong et al. 2017 | Conference Proceeding |
| 2 | Bateman et al. 2016 | Conference Abstract |
| 3 | Hambling et al. 2016 | Position statement |
| 4 | Nyugen et al. 2015 | Conference Abstract |

Supplementary Table 3: Potential Source of conflict of interest and funding

| **Study** | **Year** | **Conflict of Interest** | **Source of Funding** |
| --- | --- | --- | --- |
| Wang et al.(17) | 2023 | No | None |
| Huang et al. (18) | 2023 | No | Ministry of Science and Technology, Taiwan |
| Weng et al.(19) | 2023 | No | Taichung Veterans General Hospital, Taichung  Taiwan Ministry of Science and Technology |
| Mickute et al.(20) | 2023 | No | NIHR Leicester Biomedical Research Centre, which is a partnership between the University Hospitals of  Leicester NHS Trust and the University of Leicester. |
| Lin et al.(21) | 2023 | No | Wenzhou Science and Technology Bureau |
| Wu et al.(22) | 2022 | No | National Key Research and Development Program of China (2020YFC2006300) and the National Natural Science Foundation of China (82003461). |
| He et al.(23) | 2022 | No | Zhejiang Provincial Public Welfare  Fund Project of China (GF21H260034), the Key Laboratory of Intelligent  Preventive Medicine of Zhejiang Province (2020E10004), and National  Natural Science Foundation of China (82171584). |
| Espeland et al.(24) | 2022 | No | National Institute of Diabetes and Digestive and Kidney Diseases, National Institutes of Health, Department of Health and Human Services |
| Akan et al.(25) | 2022 | No | None |
| Leung et al.(26) | 2021 | No | National Institute on Aging and the National Institutes of Health |
| Presley et al. (27) | 2019 | No | Veterans Affairs Clinical Science Research and Development [CX000570-06 to C.L.R.]; by National Institutes of Health – National Institute on Aging [R01AG043471 to C.G.G]; by Vanderbilt Center for Diabetes Translation Research [P30DK092986 to C.L.R.] |
| Kitamura et al. (28) | 2019 | No | Ministry of Education, Culture, Sports, Science and Technology, Japan |
| Ferri-Guerra et al. (29) | 2020 | No | Miami VA Healthcare System GRECC |
| Chao et al. (30) | 2018 | No | National Taiwan University Hospital BeiHu branch, and the Ministry of Science and Technology, Taiwan |
| Li et al. (31) | 2018 | No | Ministry of Science and Technology, Taiwan. |
| Thein et al. (32) | 2018 | No | Agency for Science Technology and Research  (A*STAR) Biomedical Research Council (BMRC) [Grant: 08/1/21/19/567] and from the National Medical Research Council [Grant: NMRC/1108/2007] |
| Castro-Rodriguez et al. (33) | 2016 | No | Supported by grants RD12/43, PI031558, PI07/90637, and PI10/01532 (Instituto de Salud Carlos III, Spain), FSE/FEDER, MIDFRAIL (no: 278803), and FRAILOMIC (no: 305483) EU FP7-HEALTH Projects |
| Chode et al. (35) | 2016 | No | National Institute on Aging |
| Liccini et al. (34) | 2016 | No | Aging Research Grant sponsored by the American Federation of Aging Research. |
| Li et al. (36) | 2015 | No | Key Technology Research & Development Program of Sichuan Province (2013ZR0163, 2013FZ0090) and Health  and family planning commission of Sichuan province (Chuanganyan 2012-106). |
| Wang et al. (37) | 2014 | No | National Institute of Diabetes and Digestive and Kidney Diseases K25DK075092 and National Cancer Institute R21CA161180 |
| Cacciatore et al.(38) | 2013 | No | Not Mentioned |
